# Highly‐Scattering Cellulose‐Based Films for Radiative Cooling

**DOI:** 10.1002/advs.202104758

**Published:** 2022-01-17

**Authors:** Juliana Jaramillo‐Fernandez, Han Yang, Lukas Schertel, Guy L. Whitworth, Pedro D. Garcia, Silvia Vignolini, Clivia M. Sotomayor‐Torres

**Affiliations:** ^1^ Catalan Institute of Nanoscience and Nanotechnology (ICN2) CSIC and BIST Campus UAB, Bellaterra Barcelona 08193 Spain; ^2^ Department of Chemistry University of Cambridge Lensfield Road Cambridge CB2 1EW UK; ^3^ ICREA—Institució Catalana de Recerca i Estudis Avançats Barcelona 08010 Spain; ^4^ Present address: The Institute of Photonic Sciences (ICFO) Mediterranean Technology Park Avinguda Carl Friedrich Gauss, 3 Castelldefels Barcelona 08860 Spain; ^5^ Present address: School of Chemical Engineering University of Chinese Academy of Sciences Beijing 100049 China

**Keywords:** cellulose, high mid‐infrared emittance, low solar absorption, radiative cooling, scattering

## Abstract

Passive radiative cooling (RC) enables the cooling of objects below ambient temperature during daytime without consuming energy, promising to be a game changer in terms of energy savings and CO_2_ reduction. However, so far most RC surfaces are obtained by energy‐intensive nanofabrication processes or make use of unsustainable materials. These limitations are overcome by developing cellulose films with unprecedentedly low absorption of solar irradiance and strong mid‐infrared (mid‐IR) emittance. In particular, a cellulose‐derivative (cellulose acetate) is exploited to produce porous scattering films of two different thicknesses, *L* ≈ 30 µm (thin) and *L* ≈ 300 µm (thick), making them adaptable to above and below‐ambient cooling applications. The thin and thick films absorb only ≈5% of the solar irradiance, which represents a net cooling power gain of at least 17 W m^−2^, compared to state‐of‐the‐art cellulose‐based radiative‐cooling materials. Field tests show that the films can reach up to ≈5 °C below ambient temperature, when solar absorption and conductive/convective losses are minimized. Under dryer conditions (water column = 1 mm), it is estimated that the films can reach average minimum temperatures of ≈7–8 °C below the ambient. The work presents an alternative cellulose‐based material for efficient radiative cooling that is simple to fabricate, cost‐efficient and avoids the use of polluting materials.

## Graphical abstract

Thin cellulose‐based films are prepared via phase separation with unprecedented scattering efficiency in the UV–visible range (down to 300 nm), that radiate heat in the mid‐infrared, and exhibit radiative cooling. Field tests show that the films can reach up to 5 °C below ambient temperature, when solar absorption and conductive/convective losses are minimized. They are cost‐effective and environmentally friendly.

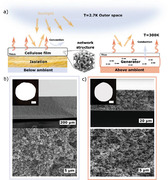

## 1 Introduction

The Earth keeps its thermal equilibrium by dissipating the excess heat from absorbed solar energy to the cold outer space (≈3 K) via thermal radiation. This heat exchange between the planet's surface and outer space takes place because the atmospheric gases barely absorb the outgoing long‐wave radiation between 8 and 13 µm. This spectral band, known as the first infra‐red (IR) atmospheric transparency window (IRAW), coincides with the peak wavelength (9.7 µm) of black‐body radiation at average ambient temperature (≈300 K), enabling the maximization of radiative heat transfer. The same principle applies to all the objects on the Earth's surface. When exposed to the sky, objects radiate the excess heat to outer space through the IRAW^[^ ^1^, ^2^, ^3^ ^]^ and exchange heat with the atmosphere at wavelengths where both the objects and atmosphere are opaque. To some extent, the release of heat is damped due to the absorption of the outgoing long‐wave radiation by the atmospheric gases at wavelengths outside the IRAW. The absorption by the atmosphere and the consequent radiation of this energy in the infrared (by Kirchhoff's law of thermal radiation)^[^ ^4^ ^]^ results in further heating the object at the Earth's surface. Thus, its net radiative heat flux to outer space is limited by the incident thermal radiation from the atmosphere and the absorbed sunlight. Radiative cooling technologies exploit this phenomenon to achieve passive sub‐ambient cooling during daytime, and have emerged recently as a promising solution in renewable‐energy research.

To achieve efficient radiative cooling, the optical properties of materials must be engineered to i) minimize the absorption of sunlight and atmospheric thermal radiation and ii) enhance their emittance within the atmospheric transparency window. Many such materials have now been demonstrated using nano‐ and micro‐structured technologies and surface engineering, promising a viable cooling technology for intensive global energy savings that could help mitigating climate change.^[^ ^5^, ^6^, ^7^, ^8^, ^9^, ^10^ ^]^ For example, remarkable designs include microsphere‐based photonic random media,^[^ ^11^ ^]^ thin‐film multi‐layer structures,^[^ ^12^, ^13^, ^14^, ^15^, ^16^ ^]^ microsphere‐periodic arrays,^[^ ^17^, ^18^ ^]^ metal–dielectric nanophotonic structures,^[^ ^19^ ^]^ double‐layer nanoparticle‐based coatings,^[^ ^20^, ^21^ ^]^ aerogels,^[^ ^22^, ^23^ ^]^ porous synthetic polymer‐based coatings,^[^ ^24^, ^25^ ^]^ and hybrid dielectric–polymer materials.^[^ ^26^, ^27^, ^28^, ^29^, ^30^ ^]^ However, several of these technologies come at the cost of demanding engineering and fabrication processes, making difficult their large scale production and therefore their use in real applications. Furthermore, one would ideally exploit sustainable materials to produce such coatings and avoid the use of plastics or heavy metals, to have a meaningful positive impact on the environment. In this context, cellulose derivatives are ideal candidates as they can be produced from cellulose in industrial scale by various chemical modifications.^[^ ^31^ ^]^ Specifically, cellulose acetate (AC) is an insoluble cellulose derivative considered as a nontoxic and biodegradable material.^[^ ^32^, ^33^ ^]^ Here, we report on producing highly scattering porous cellulose films for efficient radiative cooling with a scalable production process that does not require any particular or challenging nanostructuring or engineering.

## 2 Results and Discussion

Thin films of cellulose (acetate) with a disordered network morphology were produced by a phase separation method. The achieved porosity enables strong light scattering down to UV wavelengths and across the visible solar spectrum (Figure 2b), while showing thermal emission in the mid‐IR and the IR atmospheric transparency window. We characterize the network morphology, optical transmittance and reflectance spectra as well as their thermal properties to show their applicability and performance for radiative daytime cooling and discuss their potential for sustainable large scale coating applications.

**Figure 1:**
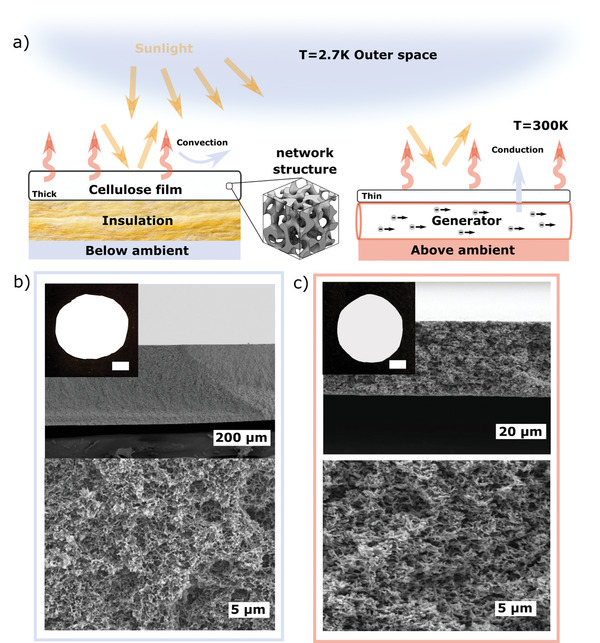
Highly scattering cellulose‐based radiative cooling films. a) Illustration of radiative below‐ambient (right) and above‐ambient (left) cooling applications for a thick and a thin cellulose‐based film. The inset is a schematic representation of the disordered network scattering structure. Sunlight (yellow) is reflected by the network structure of the film while thermal radiation (red) is emitted. The thin film enables improved heat conduction, favorable for above‐ambient cooling applications while the thick one provides thermal insulation from conduction and convection. Electron microscopy images of b) a thick (≈300 µm, filling fraction =48%) film and c) a thin (≈30 µm,, filling fraction =36%) film made of cellulose acetate (AC). Top: Cross‐section image. Scale‐bars are 200 and 20 µm. Bottom: Zoom in. Scale‐bars are 5 µm Inset: Macroscopic image of the films. Scale‐bars are 1 cm.

**Figure 2:**
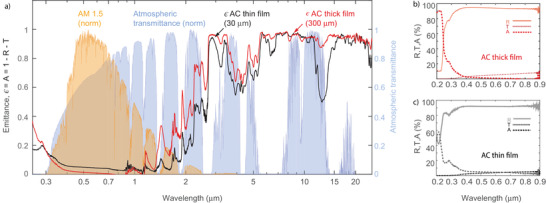
Optical properties of the cellulose‐based radiative cooling films in the visible and mid‐IR. a) Comparison of the spectral emittance, ϵ, of the thin (black) and thick (red) cellulose based films measured from 0.25 to 25 µm. The spectra superimposed depict the normalized mid‐latitude solar irradiance with an air mass coefficient (AM) of 1.5^[^ ^36^ ^]^ (orange) and atmospheric transmittance from MODTRAN^[^ ^37^ ^]^ (blue). Reflectance, transmittance and absorptance of b) thick and c) thin cellulose‐based films in the UV–visible, from 0.2 to 0.9 µm

### 2.1 Tunable Thermal and Optical Properties of Radiative Cooling Films

Passive radiative cooling materials must emit heat to the ultracold‐outer space through the IRAW (λ ≈ 8–13µm). Only when the emitted power exceeds the power absorbed by the material over all other wavelengths, the material is able to cool below ambient temperatures. During daytime, achieving temperatures below ambient can be challenging due to heating of the structures by incident sunlight. The performance of passive‐daytime radiative cooling (PDRC) materials with high emittance in the IRAW can be therefore enhanced by designing their morphology to minimize absorption of solar irradiance (i.e., reflecting it optimally) over a wide spectral range (λ ≈ 0.3–2.5µm). Such broadband high reflectance is usually easily achieved in thick films of random‐network structures through multiple light scattering.^[^ ^34^ ^]^ However, this is not trivial in thin films because the light transport mean free path (MFP), which is the length scale on which the orientation of the incoming light is randomized by scattering, is comparable or greater that the film thickness *L*. On a microscopic length scale, the scattering is limited by the ratio of the film thickness *L* and the MFP. Thus, achieving high broadband reflectance in thin‐films is difficult as the MFP in nonoptimized scattering materials is usually several microns long.^[^ ^35^ ^]^

The thickness of the porous PDRC material is an important design parameter because it not only affects the optical properties but also the thermal performance. Films with larger thickness *L* can be advantageous to easily achieve strong emittance in the infrared and, at the same time, to provide thermal insulation to minimize parasitic conduction losses. Such characteristics are useful for applications that require cooling below the ambient‐temperature (below‐ambient cooling). On the other hand, thin structures can be more beneficial for above‐ambient cooling, where heat conduction is favorable, for example, when the substrate emits a lot of heat, such as in a generator (**Figure** **1**a).

Here, only using one single approach we produce, via phase‐separation, films of cellulose (acetate) with different thicknesses and controlled scattering morphology. We show that the produced thick films (≈300 µm, filling fraction =48%) strongly reflect the sunlight and emit in the mid‐infrared (mid‐IR) while can also provide thermal insulation to the underlying substrate. On the other hand, the thin films (≈30 µm, filling fraction = 36%) allow partial transmittance and moderate emittance in the IRAW while still strongly reflecting in the solar spectrum. Figure 1b,c show scanning electron micrographs of the films from which the thickness and morphology were characterized (see Experimental Section for details).

### 2.2 Optical Characterization

To maximize their cooling effect, we need to engineer the optical properties of radiative cooling materials over a large spectral range from the UV to the mid‐IR. In **Figure** **2**, the optical properties of a thin (black) and a thick (red) film are shown and their emittance is plotted in Figure 2a. For comparison, we also plot the normalized solar irradiance spectrum at air mass (AM) 1.5 (yellow) and the atmospheric transmittance (blue). The features observed in both thick and thin films are very similar over the entire spectral range. A major difference lays in the lower emittance of the thin film around 12 µm which is directly associated to a higher transmittance. This could enable the use of our materials not only for protection and cooling in below‐ambient applications but also for above‐ambient cooling, that is, heat generating substrates. The optical properties of the thick and thin films in the ultraviolet (UV)/visible (vis) spectra are plotted in Figure 2b,c, respectively. Both films show a low absorption down to 300 nm. It is noteworthy that both films have a remarkable high reflectance over the entire visible range and absorb only about 5% of the solar irradiance, even in the case of the thin film (**Table** **1**). The high reflectance in the visible is mainly caused by an optimal pore size distribution for Mie scattering with dominating sizes in the hundreds of nanometers (Figure S1, Supporting Information). The low absorptance combined with the high‐broadband reflectance over the whole solar spectrum makes these structures ideal for passive daytime radiative cooling.

**Table 1:** Comparison of the absorptance in the UV–vis or mid‐IR spectral ranges for recently reported cellulose‐based materials for radiative cooling. The symbol “‐” refers to data not available

	Thickness	Solar abs.	Solar abs.	ϵ_IRAW_	T_IRAW_	ϵ_mid‐IR_
	[µm]	power [W m^−2^]	[%]	[%]	[%]	[%]
		(0.3–2.5 µm)	(0.3–2.5 µm)	(8–13 µm)	(8–13 µm)	(2.5–25 µm)
AC thin film	30.0	46.7	5.2	87.9	7.5	78.2
AC thick film	300.0	44.8	5.0	93.6	1.3	87.1
Hybrid structural material ^[^ ^41^ ^]^	4000.0	64.2	7.2	91.1	–	85.9
Structural material ^[^ ^38^ ^]^	⩾10 000.0	70.4	7.8	91.7	–	86.1

### 2.3 Radiative Cooling Performance

**Figure 3:**
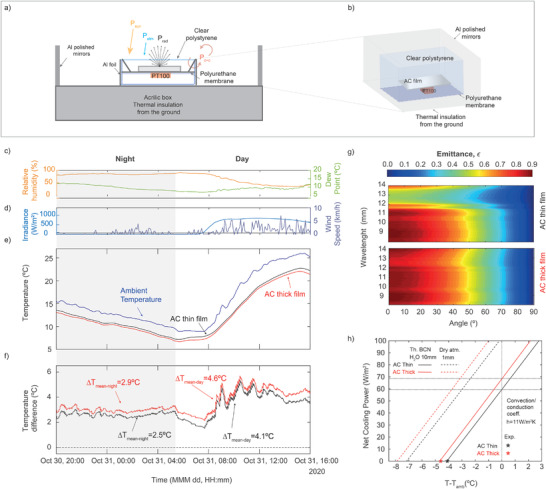
Testing the radiative cooling performance of cellulose‐based films. a) Cross section of the cellulose‐based films in the test set‐up insulated from the ground for continuous temperature measurements. b) Schematics of the test setup. c–g) Continuous temperature measurement for the thin and the thick AC films, along with the environmental conditions. c) Relative humidity (RH) (yellow curve, left axis) and dew point (green curve, right axis). d) Solar irradiance (blue curve, left axis) and wind speed (purple curve, right axis). e) Measured cycle of temperature during 24 h in a clear‐sky day for the thin and the thick AC films. The ambient temperature is plotted in blue for reference. f) Temperature difference between the ambient and the AC films. g) Spectral directional emittance of thin (top) and thick (bottom) cellulose‐acetate nanostructured films, assuming that they behave as Lambertian emitters. h) Predicted net cooling power of the thin and thick cellulose‐based films, for a conduction/convection coefficient of 11 Wm^−2^K^−1^, high atmospheric water vapor content (water column =10 mm), representative of a Mediterranean location, such as Barcelona (continuous lines) and dry conditions (water column =1 mm) (dashed lines).

The radiative cooling of the cellulose‐based films was evaluated by measuring the temperature reached by both films and comparing that with the ambient temperature. The films were attached to PT100‐thermometers and the ambient temperature was measured using a Bresser PC weather station probe. A schematic illustration of the experimental test set‐up and a cross‐section depicting the heat exchange processes are shown in **Figure** **3**a,b. Details are given in the Experimental Section. The thermal measurements were performed by exposing the experimental setup to a clear sky throughout a 24‐h day–night cycle at the rooftop of a four‐story building in Barcelona (Spain) in Autumn.

In Figure 3c–f, we plot the 24‐h cycle of the measured temperature of both the thin and the thick cellulose‐based films along with the environmental conditions during the measurement. For reference, Figure 3c shows the relative humidity (RH) (yellow curve, left axis) and the dew point (green curve, right axis), while Figure 3d shows the solar irradiance (light‐blue curve, left axis) and the wind speed (dark‐blue curve, right axis). The time evolution of the temperature from both films and their corresponding temperature difference relative to the ambient are plotted in Figure 3e,f, respectively. During daytime, both films maintained their temperatures below the ambient (Figure 3b). Average below‐ambient temperatures of 4.6 and 4.1 °C were measured for the thick and thin films, respectively, from 11:00 to 16:00, as plotted in Figure 3e. During this period, the sky was clear, with a mean solar irradiance of 780Wm^−2^, a wind speed of 2.4 km h^−1^ (Figure 3d) and an average relative humidity of 40% (Figure 3c). The maximum temperature difference with respect to the ambient was 5.3 and 4.9 °C around 11:30, for the thick and the thin film, respectively. These results indicate that the produced cellulose films are capable of both preventing heating from diffuse solar irradiance and efficiently emitting heat as IR radiation. Finally, the maximal absolute temperature reduction from ambient was 5.5 and 5.2 °C measured just before noon (at 11:28) and at peak irradiance of 840 Wm^−2^, for the thick and thin film, respectively. During the night, both films keep their temperatures below that of the ambient air, yet the temperature reduction compared to daytime is smaller, which suggest a decreased radiative cooling power. Average below‐ambient temperatures values of 2.9 and 2.5 °C were recorded for the thick and the thin film, respectively (from 20:00 to 8:00, with average relative humidity of 86% and wind speed of 0.5 km h^−1^). The reduced capability of both films to keep temperatures below that of the ambient air during the night is attributed to the higher relative humidity. The maximal temperature below ambient was 3.5 and 3.0 °C measured at 21:30, before the dew point temperature started to drop from 12 °C.

To understand the measured thermal performance of the produced cellulose films, we calculate their daytime net cooling power as a function of the temperature difference between the radiative coolers and the ambient. For this purpose, their spectral emittances measured by Fourier‐transform infrared (FT‐IR) are extrapolated for angles from 0° to 90°, assuming that they behave as Lambertian emitters. In Figure 3g, we plot the directional spectral emittance of thin (top) and thick (bottom) films, over the IR transparency atmospheric window (from 8–14 µm). In Figure 3h, we report the net cooling power estimated using the emittance values over the full mid‐IR spectrum, considering a convection and conduction coefficient of *h* = 11 W m^−2^K^−1^ and *T*_amb_ = 300 K. It is important to consider that to estimate the solar absorption, we only take into account diffuse sunlight, as the samples were characterized using a radiation shield. The atmospheric transmittance used to calculate the net cooling power was extracted using MODTRAN,^[^ ^37^ ^]^ for vertical water vapor column conditions typical of Barcelona (estimated water column =10 mm), that is, high content of total gaseous water in a vertical column of atmosphere, compared to dry conditions (more details in Experimental Section). The results are plotted in Figure 3h, as black (thin film) and red (think film) continuous lines. For the sake of comparison, we also report the theoretical net cooling power that can be achieved under dry atmospheric conditions (water column =1 mm), as dashed lines in Figure 3h. Finally, the average minimum temperature of the film achieved experimentally (in the steady‐state regime) is shown in Figure 3h, as open black (thin film) and red pentagons (thick film), respectively. This equilibrium temperature is reached when the net cooling power becomes equal to zero, $T_{r|P_{\text{cool}}=0}$. We use the difference between the emitter's temperature and the ambient at this point to characterize the below‐ambient cooling performance of the surfaces (more details in Experimental Section).

The black and red stars in Figure 3h pinpoint the average minimum temperatures measured below ambient in the thick and thin films, 4.1 and 4.6 °C, respectively, which match our predictions. The location of the experiment, close to Barcelona, is characterized by a Mediterranean climate, which exhibits higher water vapor content compared to other arid or high altitude locations under which several daytime radiative cooling experiments have been reported in the literature.^[^ ^5^, ^22^, ^24^, ^38^ ^]^ The water vapor content in the atmosphere affects the radiative cooling efficiency which is reduced due to the absorption of the heat emitted from the film by the water vapor molecules in the lower atmosphere. We expect our films to reach average minimum temperatures of 7 (thin film) and 8 °C (thick film) below the ambient under dryer conditions (water column =1 mm), as indicated in Figure 3h by including a correction with dry conditions (dashed curves). For negligible convection and conduction (coefficient *h* = 0 W m^−2^K^−1^), our model predicts average minimum cooling temperatures of 37 (thin film) and 39 °C (thick film) relative to the ambient under dry atmospheric conditions. This requires to completely eliminate the non‐radiative losses, as in previously reported works, for example, refs. [39, 40].

### 2.4 Toward Scalable and Sustainable Radiative‐Cooling Coatings

Hybrid cellulose‐based materials have been designed recently to optimize their optical and thermal performance for radiative cooling in either the vis or mid‐IR spectral regimes.^[^ ^27^, ^41^, ^42^, ^43^, ^44^ ^]^ In these materials, cellulose is serving as a matrix and/or scattering material to optimize the optical properties in the visible but the IR emission is obtained by adding thermal‐emitting polar materials such as Al_2_O_3_, TiO_2_, and SiO_2_ nano or microspheres. Here, we engineer cellulose as an all‐in‐one radiative‐cooling material by obtaining thick and thin films via phase separation with optimized optical and thermal properties simultaneously. Our material has been additionally tested for radiation durability using a Bandol wheel instrument in which the films were irradiated for 100 h, which is equivalent to 50–100 years of indoor illumination conditions, or about 6 months of direct outdoor sun exposure. The color change (△*E*_00_) is a useful number for assessing subtle changes. For reference, humans in general would not visually notice a difference until △*E*_00_ reaches ≈2. The △*E*_00_ of the thin and thick AC films after Bandol wheel test is only 0.88 and 0.16, respectively.

A comparison of the physical properties relevant to radiative cooling between recently reported cellulose‐based materials and our films is shown in Table 1. Chen et al.^[^ ^41^ ^]^ and Li et al.^[^ ^38^ ^]^ were the first to report on cellulose‐based structural materials for radiative cooling with excellent mechanical properties for architectural applications. These materials were developed for building applications and therefore their dimensions are, at least, one order of magnitude greater than the films here reported. Interestingly, the highly scattering AC films absorb a much lower fraction of solar irradiance, as cellulose acetate has better performance in the UV spectral range compared to pure cellulose. The heating power resulting from solar absorption is estimated to be ≈47 and 45 W m^−2^ for the thin and the thick films, respectively, compared to 64 and 70 W m^−2^ for the thicker materials from refs. [41] and [38]. This reduction represents a gain in net cooling power of at least 17 W m^−2^, despite the fact that our films are two and one order of magnitude thinner than the reported structural materials. Very recently, Gamage et al.,^[^ ^45^ ^]^ demonstrated reflective and transparent radiative coolers based on cellulose derivatives manufactured via electrospinning and casting. Our 300 µm‐thick films exhibit comparable optical properties in the visible and mid‐IR with respect to their 275 µm‐thick film. In contrast, our 30 µm‐thin films achieve high reflectance across the whole solar spectrum and strong average IRAW emittance (Table 1), despite their small thickness, while their thinnest reported film (60 µm) is partially transparent in both wavelength ranges. This highlights the efficient broadband light scattering that we achieve in such thin films with the network scattering nanostructure.

## 3 Conclusion 

In conclusion, we have obtained efficient radiative‐cooling films using only an abundant and biodegradable cellulose‐derivative. Our scalable‐production process is based on air‐drying, which does not require any sophisticated nanostructuring neither the incorporation of dielectric particles that act as optical scatterers. The cellulose‐acetate films have a disordered network morphology enhancing the reflectance across the whole solar spectrum (λ ≈ 0.3–2.5µm), which results in minimal absorption of ≈5% of the solar irradiance (Table 1). At the same time, the produced films exhibit strong mid‐IR emittance, in particular within the IR atmospheric transparency window. In addition, we demonstrated that by tuning the thickness, different optical and thermal properties can be achieved: The thick films have stronger emittance in the IR atmospheric transparency window ($\varepsilon_{\text{IRAW}}\approx 93.6\%$) and are suitable for sub‐ambient applications, where conduction and convection minimization is required to cool down an underlying substrate. The thin films exhibit slightly lower $\varepsilon_{\text{IRAW}}\approx 87.9\%$ and modest transmittance $T_{\text{IRAW}}\approx 7.5\%$ in the IR atmospheric transparency window. Their reduced thickness (lower thermal resistance), makes them ideal for above‐ambient applications, where the underlying material produces heat and convective/conductive heat transfer become beneficial for the net cooling (see the Supporting Information). All the thermal performances of the cellulose‐based radiative coolers were assessed during field tests. The produced thin and thick cellulose films reached up to ≈5.0°C and ≈5.2°C below ambient temperature, when convection/conduction losses and direct solar irradiance where minimized. Under dryer conditions (water column = 1 mm), we estimate that our films can reach average minimum temperatures of 7 (thin film) and 8 °C (thick film) below the ambient. We, therefore, believe that our work provides a novel all‐in‐one large‐scale cost‐efficient radiative cooling material fabricated using solely sustainable cellulose‐based compounds, that are adaptable to different types of cooling applications.

## 4 Experimental Section

### Materials

Cellulose acetate—Acetylcellulose—(AC) with relative molecular mass ≃ 29 000) and acetone were purchased from Sigma Aldrich.

### Preparation of Cellulose Acetate Thin Films

The films were prepared via air drying in a petri dish from the same cellulose acetate solutions containing acetone, water, and cellulose acetate powder. About 3% wt. cellulose acetate was added to an acetone–water mixture solution with 5% wt. of water. The suspension was stirred vigorously to completely dissolve the cellulose acetate powder. The final solution contained 3% cellulose acetate. Thin white films were formed by slowly drying the cellulose acetate–acetone–water mixture solution in a glass petri dish.

### Preparation of Cellulose Acetate Thick Films

Cellulose acetate powder was added into pure acetone under vigorously stirring till its weight percentage was 24% wt. The glue‐like solution was transferred into a glass petri dish which was soaked in water to form a white film and dried at 60 °C in a vacuum oven. The thickness of the film was determined by the total volume of the solution poured into a petri dish with a diameter of 4.7 cm. Typically, 1 and 2 mL solutions were used for thin and thick films, respectively.

### Characterization of Cellulose‐Based White Films by scanning Electron Microscope

The cross‐section of each film was measured by scanning electron microscope (SEM) with a Mira3 system (TESCAN) operated at 5 kV and a working distance of about 6 mm. To prepare specimens, the films were frozen in liquid nitrogen and then cracked. The samples were mounted on aluminum stubs using conductive carbon tape and coated with a layer of platinum (10 nm in thickness) by a sputter coater (Quorum Q150T ES). The thickness of each film was determined from the SEM images of their cross‐sections.

### Optical Characterization of Cellulose‐Based White Films by UV–Vis Spectroscopy

The optical properties of the films in the UV/vis spectra where measured from 300 to 800 nm using a Cary 4000 spectrometer equipped with an integrating sphere.

### Characterization of Cellulose‐Based White Films by FT‐IR

The emittance of the films in the mid‐IR was measured by FT‐IR spectroscopy, using a gold integrating sphere to collect the near‐normal reflected, transmitted, and scattered light over a solid angle of 2π sr, from wavelength of 0.9 to 25 µm. A direct approach to obtain the emittance was by measuring the absorptance *A*, the fraction of radiation absorbed by the films. It was assumed that the directional spectral emittance was equal to the directional spectral absorptance, ϵ(λ, θ, ϕ) = *A*(λ, θ, ϕ) under thermodynamic equilibrium, according to the Kirchhoff's law of thermal radiation.^[^ ^46^ ^]^ The absorptance was given directly by taking the difference of the measured reflectance and transmittance of the samples in the infrared spectrum from unity (*A* = 1 − *R* − *T*).

### Atmospheric Transmittance and Net Cooling Power Calculations

The atmospheric transmittance used in the net cooling power calculation was obtained using moderate resolution atmospheric transsmission (MODTRAN).^[^ ^37^ ^]^ MODTRAN is a computer code used to predict atmospheric spectral transmittances and radiances over the ultraviolet through long wavelength infrared spectral regime. The atmosphere model considered a mid lattitude region during summer, which corresponded to a vertical water vapor column conditions typical of Barcelona, that is, high content of total gaseous water in a vertical column of atmosphere, compared to dry conditions. The net cooling power calculations were performed following the procedure described in ref. [17].

### Continuous Passive Radiative Cooling Measurements of Cellulose‐Based Films

During the experiment, the parasitic conduction and convection were minimized. To reduce these heat losses, the films were placed on a low‐thermal conductivity membrane suspended inside a poly‐methacrylate box used as a sample holder and covered with aluminum foil to minimize heat gain due to solar absorption. The top of the poly‐methacrylate box was covered with a low‐density polyethylene film that was transparent in the relevant wavelengths, in order to reduce convection through the top. Further details are given in Supporting Information. The poly‐methacrylate boxes used as sample holders were placed on an acrylic structure, of 70 cm of height, that thermally insulated the system from the ground. The structure was also covered with aluminum foil to reflect the visible light and minimize parasitic heating (Figure 3b). Polished aluminum sheets were placed as solar radiation shields, that also helped to minimize the lateral convection and reflect outgoing IR radiation. The polished aluminum sheets were mainly used to avoid parasitic heating due to direct solar irradiance, as radiation shields, used following the experimental procedure reported by Chen et al.^[^ ^40^ ^]^

## Conflict of Interest

The authors declare no conflict of interest.

## Author Contributions

J.J.F, H.Y., and L.S. contributed equally to this work. S.V. and P.D.G conceived the idea and the project, and supervised the work. H.Y. fabricated the samples and performed optical measurements for material choice. L.S. analyzed the optical, the thickness, and morphology results. J.J.F. performed the UV–vis and FT‐IR spectroscopy, thermal and radiative cooling measurements, and analyzed of the optical and thermal results. G.L.W. and J.J.F calculated the net cooling power. J.J.F., P.D.G., L.S, and H.Y wrote the manuscript with all author's  contributions.

## Supporting information

Supporting Information

## Data Availability

Dataset available here: https://doi.org/10.17863/CAM.78840.
