# Supplementary material for: Highly‐Scattering Cellulose‐Based Films for Radiative Cooling
Source: Adv Sci (Weinh). 2022 Jan 17;9(8):2104758. doi: 10.1002/advs.202104758 (PMC8922136; doi:10.1002/advs.202104758)
Supplement: Supplementary file 1 — Supporting Information [file ADVS-9-2104758-s001.pdf]

ADVANCED SCIENCE
Open Access

Supporting Information

for *Adv. Sci.,* DOI: 10.1002/advs.202104758

Highly-scattering cellulose-based films for radiative cooling

Juliana Jaramillo-Fernandez, Han Yang, Lukas Schertel, Guy L. Whitworth, Pedro D. Garcia, Silvia Vignolini and Clivia M. Sotomayor-Torres

Supplementary information of

Highly-scattering cellulose-based films for radiative cooling

Juliana Jaramillo-Fernandez, Han Yang, Lukas Schertel, Guy L. Whitworth, Pedro D. Garcia, Silvia Vignolini and Clivia M. Sotomayor-Torres

A. Pore size distribution for the thin and thick cellulose-based radiative cooling films

The pore size for the thin film ($d_{avg\text{-}thin}$= 782 ± 459nm) appears larger than for the thick ($d_{avg\text{-}thick}$ =589 ± 494nm) film. This is likely caused by the difference in the drying conditions. Larger voids can form during the phase separation and solvent evaporation process if air drying is used (as for the thin film, see methods section) compared to drying in the oven (as for the thick film). Both films have a large distribution of pore sizes. However, the dominating sizes are ranging from hundreds of nm to ~1 µm. This corresponds to the optimal range for scattering visible wavelengths of light.

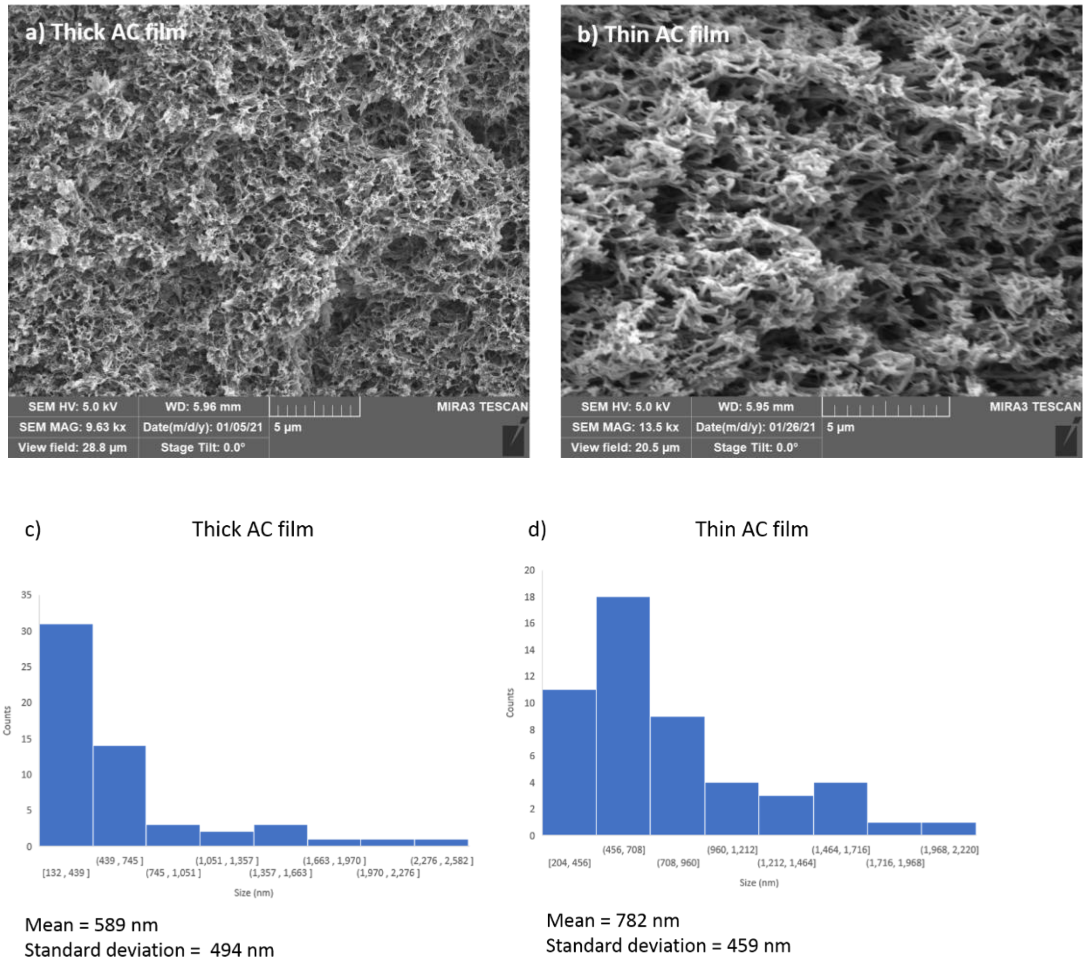

Figure S1. Comparison of the pore morphologies (SEM images) and sizes distributions for the thick (a,c) and the thin (b,d) cellulose based films, respectively.

B. Reflectance and transmittance of the AC thin and thick films, measured from 0.25 to 25 µm

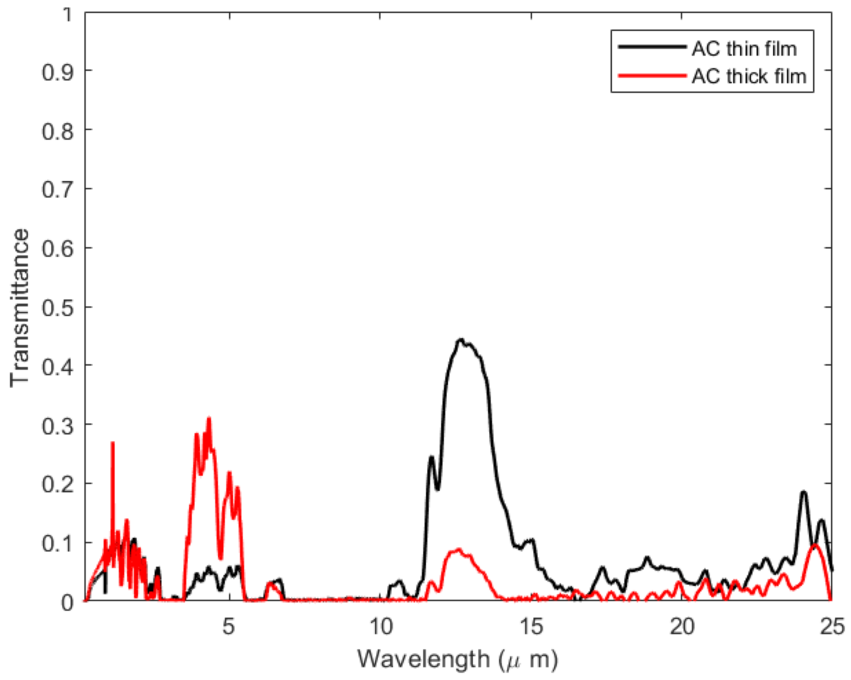

Figure S2. Comparison of the spectral transmittance, T, of the thin (black) and thick (red) cellulose based films measured from 0.25 to 25 µm.

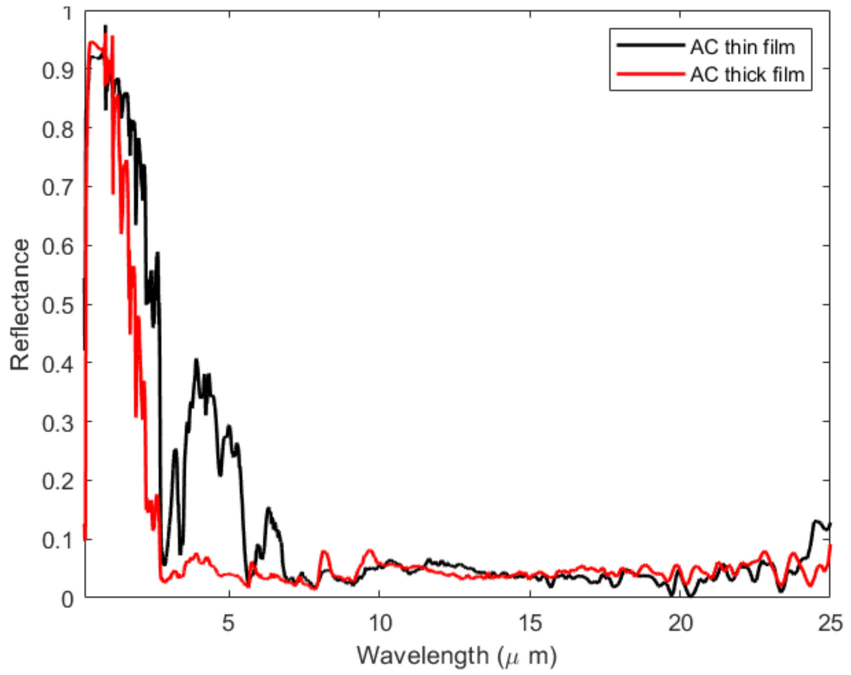

Figure S3. Comparison of the spectral reflectance, R, of the thin (black) and thick (red) cellulose based films measured from 0.25 to 25 µm.

C. Influence of the film thickness in the optical properties and radiative cooling performance

It is easier to achieve enhanced IR absorption (and thus emittance) in thick films of random-network structures through multiple scattering, compared to thin films.

This is related to the light transport mean free path (MFP), which is the length scale on which the orientation of the incoming light is randomized by scattering. The optical properties of a disordered material (defined as a system where the structure factor does not exhibit long range order) are directly determined by the optical thickness (OT). The OT is the ratio between the film thickness and the MFP. At low OT, light propagation will be partly unperturbed, resulting in light transmittance. Moreover, the MFP, is wavelength dependent. Typically, for a fixed morphology, the MFP is larger for larger wavelengths. Thus, achieving enhanced IR absorption is easier for larger film thickness.

In this work, we experimentally observe that the below-ambient cooling performance of both thick and thin film are comparable. Average below-ambient temperatures of 4.6 °C and 4.1 °C were measured for the thick and the thin films, respectively, from 11:00 to 16:00 (Fig.3e,f). These temperature values (black and red stars in Fig. 3h) match our predictions, that assume the same conduction/convection coefficient h_T = 11 W/m^{2}K for both samples. The thick film presents slightly higher reflectance in the UV-visible, and stronger emittance in the mIR, compared to the thin film (Fig. 2 and Fig.S3), which results in slightly better performance, evidenced by the results from the continuous temperature measurements (Fig.3 e,f,h). Despite its smaller thickness, the thin film exhibits excellent reflectance in the UV-visible and fair mIR emittance, resulting in comparable performance, which can be mainly attributed to its optical properties, based on the good agreement between experiments and theoretical prediction of day-time net cooling power as a function of the temperature difference between the radiative coolers and the ambient. These results highlight the efficient broadband light scattering that we achieve in such thin films with the network scattering nanostructure. Furthermore, it shows that with a thinner film, comparable cooling performance can be achieved, which has implications in the reduction of material resources and fabrication costs for future upscaling.

D. Contact angle measurements:

The water contact angle was measured by using a drop shape analysis instrument (DSA100E, Krüss) at room temperature. A water droplet of 5 µL was placed on the surface of AC films, and the contact angle was an average of five measurements on different positions of each film.

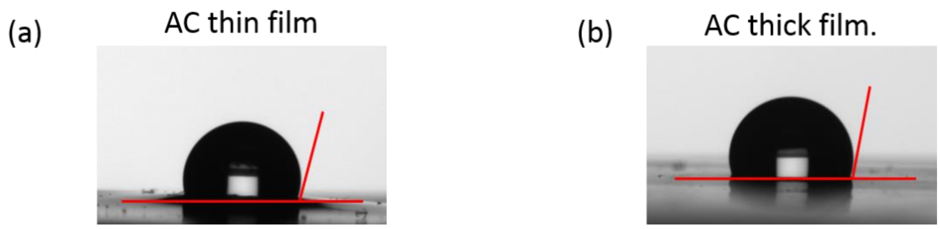

Figure S4. Contact angle measurements: image of a water drop on the (a) AC thin film, (b) AC thick film.

The results show that AC thin and thick films have a hydrophobic surface with a contact angle of 105°± 2.0° and 103°± 1.5°, respectively. No significant difference of hydrophobicity was observed between the thin and thick AC films.

E. Influence of thermal radiation *(P_r)*, solar absorption *(P_{sun})*, atmospheric absorption *(P_{atm})* and convection /conduction *(P_{c+c})* to the net cooling *(P_{net})* and radiative cooling performance

The contributions of the thermal radiation *(P_r),* solar absorption *(P_{sun}),* atmospheric absorption *(P_{atm})* and convection /conduction *(P_{c+c})* to the net cooling *(P_{net})*, depend on the combined convection and conduction coefficient, h_T, and the temperature difference between the emitter and the ambient temperature $\Delta T_{a\text{-}r}$.

To understand the role of each term to the net cooling, we calculated the different terms and relative effects in the net cooling (Figure S5), by considering a perfect broadband emitter (total directional spectral emissivity of 1 beyond 3.0 µm) that absorbs 5% of the total solar irradiance (AM 1.5). To calculate P_r (T_r) and P_{atm} (T_a) we integrate over all angles from 0 to 90, and over wavelengths ranging from 3 to 25 µm. We consider the temperature of the emitter surface T_r to vary from 200 K to 400 K, with the ambient temperature T_a being 300 K.

It is noteworthy that the effect of the non-radiative term, P_{c+c} depends on the relative temperature between the ambient and the emitter, and therefore can be beneficial or detrimental to the net cooling power, for above- or below-ambient passive cooling applications, respectively.

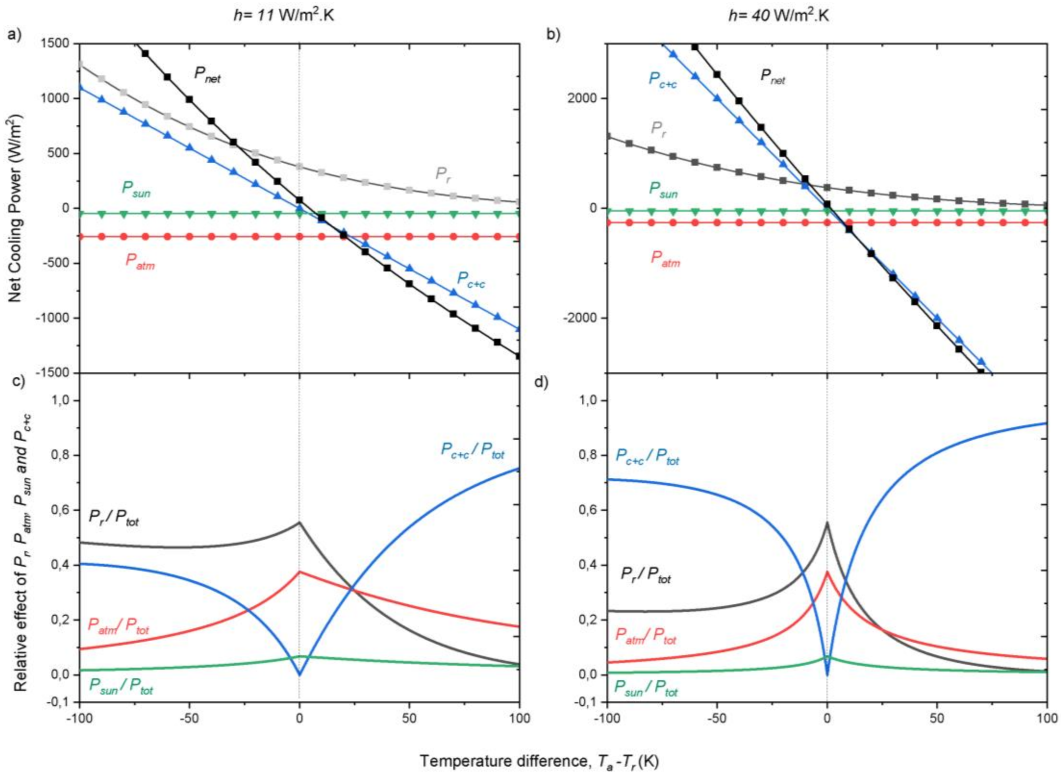

Figure S5. Contribution of each term *(P_r, P_{sun}, P_{atm}, P_{c+c})* (a),(b) and estimated relative effects on the net cooling power (c),(d), for h_T =11 and h_T =40 W/m^2.K, respectively.

Moderate convection and conduction (h_T =11 W/m^2.K), figure S5 (a),(c)

For moderate convection and conduction, the net cooling power takes negative values at $\Delta T_{a\text{-}r} \approx$ 300-295 ≈ 5 K, indicating that for greater temperature differences, an object will rather heat up instead of cooling down, because the heat gain is higher than the heat loss in the energy balance.

During our radiative cooling experiments, we approached those $\Delta T_{a\text{-}r}$ values. In that temperature regime ($\Delta T_{a\text{-}r}$ >0), the effect of the radiated power *Pr* weakens as $\Delta T_{a\text{-}r}$ increases. At $\Delta T_{a\text{-}r} \approx 5$ K, *Pr* accounts for ≈50% and Pc+c for less than ≈10% of the net cooling power. It is noteworthy, that during standard below-ambient daytime radiative cooling experiments, where $\Delta T_{a\text{-}r}$ is close to zero, conduction and convection do not play a dominant role.

However, if one aims to decrease the temperature of the emitter well below the ambient ($\Delta T_{a\text{-}r}$ > 10 K), atmospheric absorption and non-radiative losses must be suppressed. This explains why designs with high thermal insulation and selective emitters are required to achieve $\Delta T_{a\text{-}r}$ greater than 10K.

Using frequency domain thermoreflectance, we have measured the thermal conductivity of the cellulose-based films, which was found to be $k \approx 0.4 \pm 0.11 \frac{W}{\mathrm{m}.K}$. This value is in agreement with previously reported thermal conductivities of porous cellulose-based films [5].

Knowing the film thermal conductivity, we can calculate the thermal resistance, $\boldsymbol{R_{cond}}$, of the thin and the thick film and their contribution to the combined non-radiative coefficient h_T.

$$\boldsymbol{h_T = \frac{1}{R_T} = \frac{1}{R_{cond}} + \frac{1}{R_{conv}}}$$

Furthermore, the thermal resistance, is a function of the film thickness, *L*:

$$\boldsymbol{R_{cond} = \frac{L}{k}}$$

Thus, the thermal resistance is one order of magnitude greater for the thick film compared to the thin:

$$\boldsymbol{R_{cond-thin} = 7.5x10^{-5}\frac{m^2K}{W}}; \qquad \boldsymbol{R_{cond-thick} = 7.5x10^{-4}\frac{m^2K}{W}}$$

Considering, the convective coefficient h_{conv} = 11 W/m^2.K:

$$\boldsymbol{R_{T-thin} = 0.09\frac{m^2K}{W}}; \qquad \boldsymbol{R_{T-thick} = 0.0916\frac{m^2K}{W}}$$

Thus,

$$\boldsymbol{h_{T-thin} = 10.99\frac{W}{m^2K}}; \qquad \boldsymbol{h_{T-thin} = 10.90\frac{W}{m^2K}};$$

Finally, for a $\Delta T_{a\text{-}r}$=5 K, the non-radiative losses are:

$$\boldsymbol{\Delta T_{a-r} h_{T-thin} = 54.95 \frac{W}{m^2}}, \qquad \boldsymbol{\Delta T_{a-r} h_{T-thin} = 54.5 \frac{W}{m^2}}$$

The difference is 0.5 W/m^2. When the equilibrium temperature of the emitter, is close to the ambient temperature (-10 K < $\Delta T_{a\text{-}r}$ < 10 K), the contribution of the thermal resistance is negligible.

However, for above-ambient applications ($\Delta T_{a\text{-}r}$ << 0 K), conduction plays an important role, as can be seen in the left part of figure S5 (b). For example, $\Delta T_{a\text{-}r}$=-200 K:

$$\boldsymbol{\Delta T_{a-r} h_{T-thin} = 2198 \frac{W}{m^2}}, \qquad \boldsymbol{\Delta T_{a-r} h_{T-thin} = 2180 \frac{W}{m^2}}$$

The difference is 18 W/m^2. This means that the net cooling can be improved by 18W/m^2 under these conditions, when using a thin film. This effect is even stronger for greater values of the convective coefficient, h_{conv}, and for film with even lower thermal conductivity (as explained below), such as aerogels, indicating the importance of properly choosing the film thickness, according to its thermal conductivity and application.

For all temperatures above the ambient $\Delta T_{a\text{-}r}$ <0K, the radiative term P_r is the strongest effect, accounting for nearly 50% of the net cooling power (Fig.S5c). In that temperature regime, the non-radiative term *Pc+c* becomes more significant as the emitter temperature increases and adds to *Pr*, which is beneficial for the net heat loss. The atmospheric absorption by the cooler is the only term that decreases the net cooling power at above-ambient temperatures and it has up to 37% influence in the total cooling at $\Delta T_{a\text{-}r}$ =0K, then its effect decreases with $\Delta T_{a\text{-}r}$ down to 10% at $\Delta T_{a\text{-}r}$ =-100 K. From Fig.S5 (a) and (c), it can thus be inferred that the radiative cooling is dominant for above-ambient applications when moderate conduction and convection are present. In addition, conduction and convection effects contribute up to 40% for $\Delta T_{a\text{-}r}$ =-100 K and therefore they should be considered in any the design for optimized thermal management in above-ambient applications.

High convection and conduction (h_T = 40 W/m^2.K), figure S5 (b),(d)

The effect of the film thickness and its thermal resistance on the net cooling power is greater for high h_{conv}. From Figure S5b, it can be inferred that as convection and conduction increase, for example due to wind velocity, they become the predominant mechanisms in the net heat exchange. For $\mathbf{h}_T$=40 W/m2.K, the net cooling is dominated by conduction and convection, which account for 65% of the effect for $\Delta T_{a\text{-}r}$<-50K. There, the radiated power is responsible for about 25% while the atmospheric absorption decreases the cooling effect by 8%.

It should be noted that as the temperature of the emitter approaches the ambient, the convection and conduction term becomes negligible (for both h_T=11 and h_T=40W/m^2.K). At Tr=Ta, the heat loss is dominated by the radiated power (55%), although atmospheric absorption also plays an important role (35%).

In the case of h_T=40W/m^2.K, the total thermal resistances of the films are:

$$\boldsymbol{R_{T-thin} = 0.025 \frac{m^2K}{W}}; \qquad \boldsymbol{R_{T-thick} = 0.026 \frac{m^2K}{W}}$$

The combined conduction/convection coefficient

$$h_{T-thin} = 40.00\frac{W}{m^2K}; \qquad h_{T-thin} = 38.46\frac{W}{m^2K};$$

For example, $\Delta T_{a\text{-}r}$=-200 K:

$$\Delta T_{a-r}h_{T-thin} = 8000\frac{W}{m^2}, \qquad \Delta T_{a-r}h_{T-thin} = 7692\frac{W}{m^2}$$

The difference is 308 W/m^2. This demonstrates that under this conditions of high h_{conv}, the thermal conductivity and the thickness of the radiative cooling film should be considered in thermal management design for optimal performance.

Furthermore, we want to stress that even though the non-radiative term dominates at high temperatures, when the conduction and convection coefficient is very high, a perfect broadband radiator can still improve the cooling performance of a system by up to 23%.

F. Influence of the optical properties of the substrate on the radiative cooling measurements

The optical properties of the underlying material can have an influence in the radiative cooling performance when the radiative cooling film is transparent or partially transparent. In our case, the thin and the thick AC films have an average transmittance of only 2.6% (Fig.2c) and 3.7% (Fig.2b), respectively, from 0.3 to 0.9 µm.

The radiative cooling measurements here reported were performed using free-standing films. In the experimental set-up, they are supported by a low-thermal conductivity PU membrane, which is covered with Al foil to reflect sunlight. The films cover nearly the totality of the surface of the PU membrane (Fig. S6). In the experiment, the incoming sunlight is primarily reflected by the AC film.

We have estimated, from the transmittance spectra (Fig.S2), that during our experiments, only about 5% of the incoming sunlight is transmitted through the RC film and will reach the PU membrane, which is covered with the Al foil and therefore will absorb a very small fraction of it, since Al reflects mostly all visible wavelengths. The portion of light that reaches the Al foil will be reflected and eventually partly absorbed by the RC film, but we estimate that the resulting heating effect is negligible, since the fraction of this absorbed light is very small compared to the reflected.

In general, for below-ambient radiative cooling designs, it is recommended to place the RC films over a thermally insulating substrate with high reflectance at wavelengths from 0.3 up to 2.5 µm. Instead, for above-ambient cooling applications, it is recommended to place the RC films over a thermally conductive substrate with high reflectance at wavelengths from 0.3 up to 2.5 µm i.e copper.

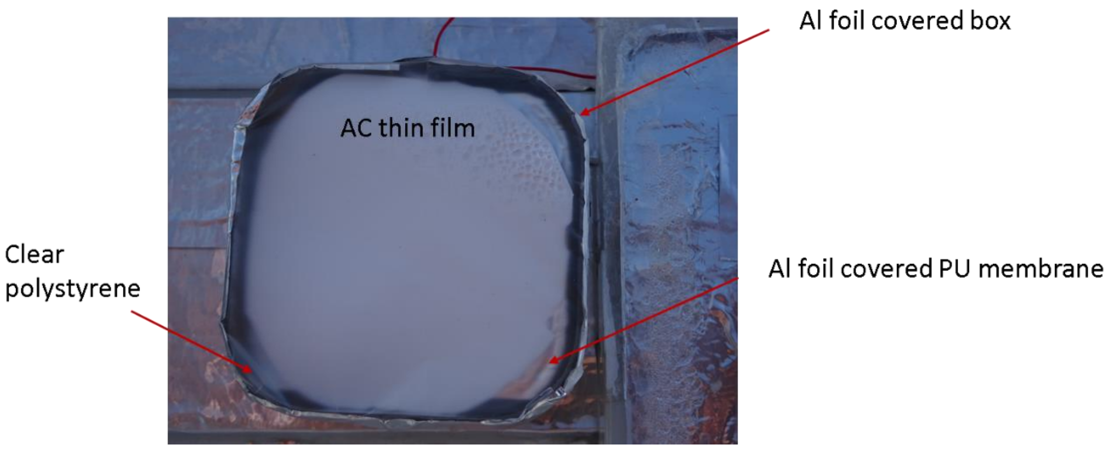

Fig.S6 Sample holder
